# Impact of storm drains on the maintenance of dengue endemicity in Presidente Prudente, São Paulo, Brazil: a geospatial and epidemiologic approach

**DOI:** 10.3389/fpubh.2024.1442622

**Published:** 2024-09-10

**Authors:** Elaine Aparecida Maldonado Bertacco, Luiz Euribel Prestes-Carneiro, Renata Ribeiro de Araújo, Lourdes Aparecida Zampieri D'Andrea, Luiza Sant'Anna Pinheiro, Edilson Ferreira Flores

**Affiliations:** ^1^Department of Statistics, School of Sciences and Technology, São Paulo State University, Presidente Prudente, São Paulo, Brazil; ^2^Municipal Epidemiological Surveillance of Presidente Prudente, Presidente Prudente, São Paulo, Brazil; ^3^Outpatient Clinic of Immunodeficiencies and Infectious Diseases, Oeste Paulista University, Regional Hospital of Presidente Prudente, Presidente Prudente, São Paulo, Brazil; ^4^Center for Biomedical Sciences and Regional Laboratory, Adolfo Lutz Institute, Presidente Prudente, São Paulo, Brazil

**Keywords:** dengue cases, outbreaks, *Aedes aegypti*, larvae, pupae, drainage system

## Abstract

**Introduction:**

Dengue is a public health challenge worldwide. Brazil registered about 70% of cases in Latin America in 2023; in 2024, the country is experiencing an unprecedented increase in the number of infected individuals. By May 2024, more than 4 million people were infected. Our goal was to: (1) determine the epidemiology of dengue cases and their spatiotemporal distribution and (2) carry out a survey of the storm drains and through a geospatial analysis to determine their possible correlation with cases of dengue in Presidente Prudente, São Paulo, Brazil.

**Methods:**

Cases and information on the habitat of mosquito in the storm drain underground drainage system from 2020 to 2021 were obtained from public agencies. Larvae, pupae, and *Ae. aegypti* were identified according to species and described in taxonomic keys. Kernel density maps were constructed.

**Results:**

From 1996 to 2023, the prevalence of cases peaked in 2016 and 2019, and in 2023 reached alarming levels, and the city was considered hyperendemic. In 2021, 2,609 cases were registered with 2 clusters of high density. Of 5,492 storm drains analyzed, 18.0% were found to have water, 9.0% had larvae or pupae of *Aedes aegypti* and 91.0% were classified as dirty or damaged. A direct correlation between the kernel layer of cases in 2021 with the kernel layer of storm drains containing water (*r* = 0.651) and larvae and pupae (*r* = 0.576) was found, suggesting that storm drains are risk factors and have an impact on the maintenance of dengue endemicity. The high number of damaged units found demonstrated the lack of storm drain management, compromising the urban drainage system and possibly contributing to dengue outbreaks.

**Conclusion:**

Policymakers may use these findings to improve existing dengue control strategies focusing on the control of storm drains and increase local and global perspectives on reducing dengue outbreaks.

## 1 Introduction

Dengue is a public health challenge worldwide, and about half of the world's population is now at risk of dengue; an estimated 100–400 million infections occur each year. Approximately 500 million people in the Americas are at risk of dengue. Brazil registers 70% of cases in Latin America in 2024, and the incidence rate has increased in recent years (1). The country is currently experiencing an unprecedented increase in dengue cases. On March 20, 2023, 489,954 probable cases were reported. On August 6, 2024, 6,449,380 probable cases were reported, with 3,176.1 cases per 100,000 inhabitants, 5,009 confirmed deaths, and a lethality rate of 0.08%. These numbers represent an increase of 1,216% in the number of cases compared with the same period in 2023 (2, 3). Recently, a change in the profile of the disease has occurred and the territory has expanded. From the north, northeast, and southwest regions, increasing levels have been found in the temperate climates of Paraná, Santa Catarina, and Rio Grande do Sul states in the south, which were free of dengue until recently (4, 5). In São Paulo, the most populous and richest area in the country, dengue is endemic throughout the state. However, in recent years, the western region was found to have a higher incidence than other regions (5). In Presidente Prudente, the most important city in that region, dengue is considered endemic.

In 2023, it had the third highest incidence in Brazil after Foz do Iguaçu and Maringa, both in Paraná state. Presidente Prudente has great vulnerability to the transmission of arboviruses, with a continuous and sustained presence of vectors, mainly *Aedes aegypti* every year. Transmission is facilitated by associated risk factors: tropical climate, rainy and dry seasons, large territorial areas, high population density, intense flow of people coming from dengue-endemic areas, as well as heterogeneity of the infrastructure, land occupation, and farming habits (4, 5).

Climate plays a significant role in the geographic expansion of dengue, affecting the life cycle of *Ae. aegypti*. Dengue vectors are known as ectotherms, i.e., their breeding and activity depend mainly on the surrounding environment (4, 5). Urbanization and population growth in urban centers in tropical developing countries of Latin America are associated with an increase in the number of dengue cases and the vector population (1). For the correct drainage of water, rainfall water-draining structures, such as storm drains, are an obligatory part of the urban landscape. However, they also may allow the development of the vector in the larvae stage and the adult stage of multiple species of resting *Ae. aegypti* and *Ae. albopictus* (6). The strategy of mapping storm drains aimed to overcome a worldwide problem that was common during the rainy season and coastal flooding (7). In Brazil, there is little information on the role of storm drains in the spread of dengue vectors and their influence on human infection.

The first cases of dengue in Presidente Prudente were detected in 1995 and the first outbreak occurred in 2006. Since then, increasing numbers of cases have been detected, and in 2022, 7.606 cases were registered. In 2023, 36,118 cases of dengue with an increase of 374.9%, and 24 deaths occurred. This was the worst year in the history of dengue and the area is considered hyperendemic. In the western region of São Paulo state, geospatial analysis has had an impact on vector-borne diseases, and vulnerable areas have been identified as a priority for public health interventions (3, 5, 8). We hypothesized whether larvae, pupae, and *Ae. aegypti* were present at the storm drains and could be correlated with the maintenance of the epidemic and outbreak of dengue in Presidente Prudente. Our goal was to: (1) determine the epidemiology of dengue cases and their spatiotemporal distribution and (2) carry out a survey analysis of the storm drains and through a geospatial analysis, determine their possible correlation with cases of dengue in Presidente Prudente, São Paulo, Brazil.

## 2 Methods

### 2.1 Study area

The western region of São Paulo state, considered endemic for dengue, comprises 45 municipalities, and in 2022, the estimated population was 744,219 (9). The region is administered by the Regional Network Health Assistance-11 (RNHA-11), located in the municipality of Presidente Prudente, the biggest and most important city in the western region. The city has the administrative headquarters of RNHA-11, the main health, education, business, and entertainment establishments, and has a large flow of people daily from the entire region. The western region is considered one of the poorest in the state and comprises sparsely populated municipalities (3, 5, 8).

A cross-sectional study was conducted between 19 August 2020 and 15 October 2021 in the urban area of Presidente Prudente (22°07′32″S, 51°23′20″W). About 15 years ago, the municipality had a typical tropical climate with a dry winter and wet summer and an average annual temperature of 23.5°C. However, due to climate change, since 2020, rainy weather with increased temperatures in the winter and dry weather with very high temperatures in the summer are common. The city is a mid-sized urban center about 560 km from the state capital, São Paulo. According to the census by the Brazilian Institute of Geography and Statistics (IBGE), in 2022 the estimated population was 225,668, inhabitants; 96% lived in the urban area. The municipality covers an area of 560,637 km^2^, and the urban area covers 16.56 km^2^; the remaining area comprises 0.668 km^2^ of Atlantic Forest and a rural area of 443.84 km^2^ (9). Its geographic location is considered a crossroads connecting São Paulo, the capital, and other states such as Mato Grosso do Sul, Paraná, and Minas Gerais. It is considered an epidemiological route of vector-borne diseases from diverse regions of Brazil and even from other South American countries such as Paraguay, Uruguay, Bolivia, and Venezuela (4, 5, 8).

**Figure 1:**
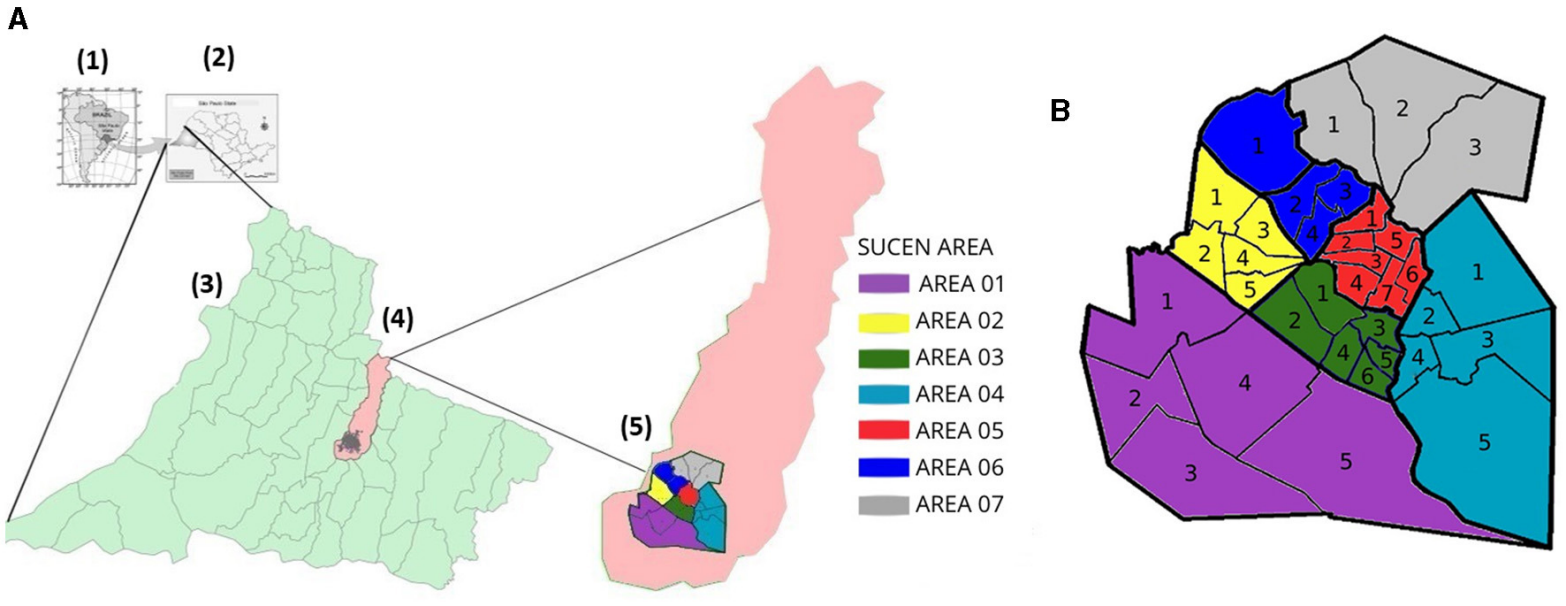
**(A)** The study setting. (1) country; (2) São Paulo state; (3) western region of São Paulo state; (4) municipality area of Presidente Prudente (5) SUCEN areas. **(B)** The seven areas are divided into different sectors.

The distribution of cases in Presidente Prudente between 1996 and 2023 was obtained from the Epidemiological Surveillance Department of Presidente Prudente. The city is divided into seven areas. Each area is composed of up to five connecting sectors, except area 03 with six sectors and area 06 with seven sectors, presenting blocks/households with similar characteristics. This division is used for epidemiologic surveillance of vector transmitted diseases (Figures 1A, B; Table 1).

**Table 1:** Dengue cases, the presence of larvae and pupae of *Ae. aegypti*, and the localization of storm drains in the urban area of Presidente Prudente.

**Area**	**Sector**	**No. of cases**	**Mean ±SD**	**95% CI**	**Larvae/pupae (n)**	**Mean ±SD**	**95% CI**	**No. of storm drains**	**Mean ±SD**	**95% CI**
1	1	63			9			2		
	2	18			^*^			^*^		
	3	49			^*^			^*^		
	4	42			0			109		
	5	20			^*^			^*^		
Total area 1		192	38.40 ± 8.61	14.47–62.33	9	4.5 ± 4.5	–/–	111	55.50 ± 53.50	–/–
2	1	8			^*^			^*^		
	2	43			^*^			^*^		
	3	40			^*^			^*^		
	4	50			^*^			^*^		
	5	61			^*^			^*^		
Total area 2		202	40.4 ± 19.83	15.78–65.02						
3	1	12			^*^			^*^		
	2	20			7			119		
	3	48			10			119		
	4	32			5			164		
	5	19			3			69		
	6	14			7			113		
Total area 3		145	24.17 ± 5.55	9.89–38.44	32	6.40 ± 1.16	3.16–9.63	584	116.8 ± 15.06	74.98–158.60
4	1	71			2			210		
	2	81			1			180		
	3	25			1			181		
	4	89			0			259		
	5	47			2			547		
Total area 4		313	62.6 ± 11.75	29.97–95.23	6	1.2 ± 0.83	0.16–2.23	1,377	275.4 ± 69.40	82.72–468.10
5	1	121			6			245		
	2	44			1			124		
	3	25			2			174		
	4	33			8			224		
	5	82			0			84		
	6	25			0			121		
	7	33			2			174		
Total area 5		363	51.86 ± 13.72	18.29–85.43	19	2.71 ± 1.16	0.14–5.57	1,146	163.7 ± 21.94	110.0–217.4
6	1	87			6			597		
	2	66			19			185		
	3	84			7			200		
	4	41			3			165		
Total area 6	52	278	69.5 ± 10.57	35.86–103.1	35	8.75 ± 3.52	2.45–19.95	1,147	286.8 ± 103.7	43.16–616.70
7	1	163			9			289		
	2	141			17			310		
	3	255			1			175		
	4	48			12			353		
Total area 7		607	151.8 ± 42.49	16.52–287.0	39	9.75 ± 3.35	0.91–30.41	1,127	281.8 ± 37.99	160.8–402.7
Total all areas	36	2,150	58.33 ± 8.16	41.75–74.91	140	5.18 ± 0.98	3.16–7.20	5,492	203.4 ± 25.14	151.7–255.1

SD, standatd deviation; CI, confidence interval; No, number of cases; (n), number of larvae/pupae. (^*^) Not available.

### 2.2 Location and surveillance of the storm drains in the urban area

Surveillance storm drains in Presidente Prudente initially resulted from the urgent need to determine the existing fauna (scorpions, *Ae. aegypti*, larvae, and venomous animals) in the storm drains. For storm drain surveillance, the city was divided into the following areas: area 01, sectors 01 and 04; area 03, sectors 02 and 06; area 04, sectors 01 to 05; area 05, sectors 01 to 07; area 06, sectors 01 to 04; and area 07, sectors 01 to 04. The survey ended in December 2021, and there was no time to cover area 2. However, the survey in area 2 is being conducted in 2024 (Figures 1A, B; Table 1).

### 2.3 Storm drain surveillance of habitats of larvae, and pupae of *Ae. aegypti*

Density in the storm drains and surveillance of habitats of larvae and pupae were performed following the Standards and Technical Recommendations for Surveillance and Control of *Ae. aegypti* in the State of São Paulo (10), and the National Guidelines for the Prevention and Control of Dengue Epidemics (11). The storm drains spreadsheet was provided by Presidente Prudente Municipality in the urban area and included 5,492 units and were surveyed during each month from Monday to Friday, from 08.00 to 11.30 h and from 14.00 to 16.00 h. If it had rained the day before the survey, the investigation was canceled and rescheduled for 2 days later. Most of the surveys were conducted in the morning.

### 2.4 Assessing the condition and classification of the storm drains

**Figure 2:**
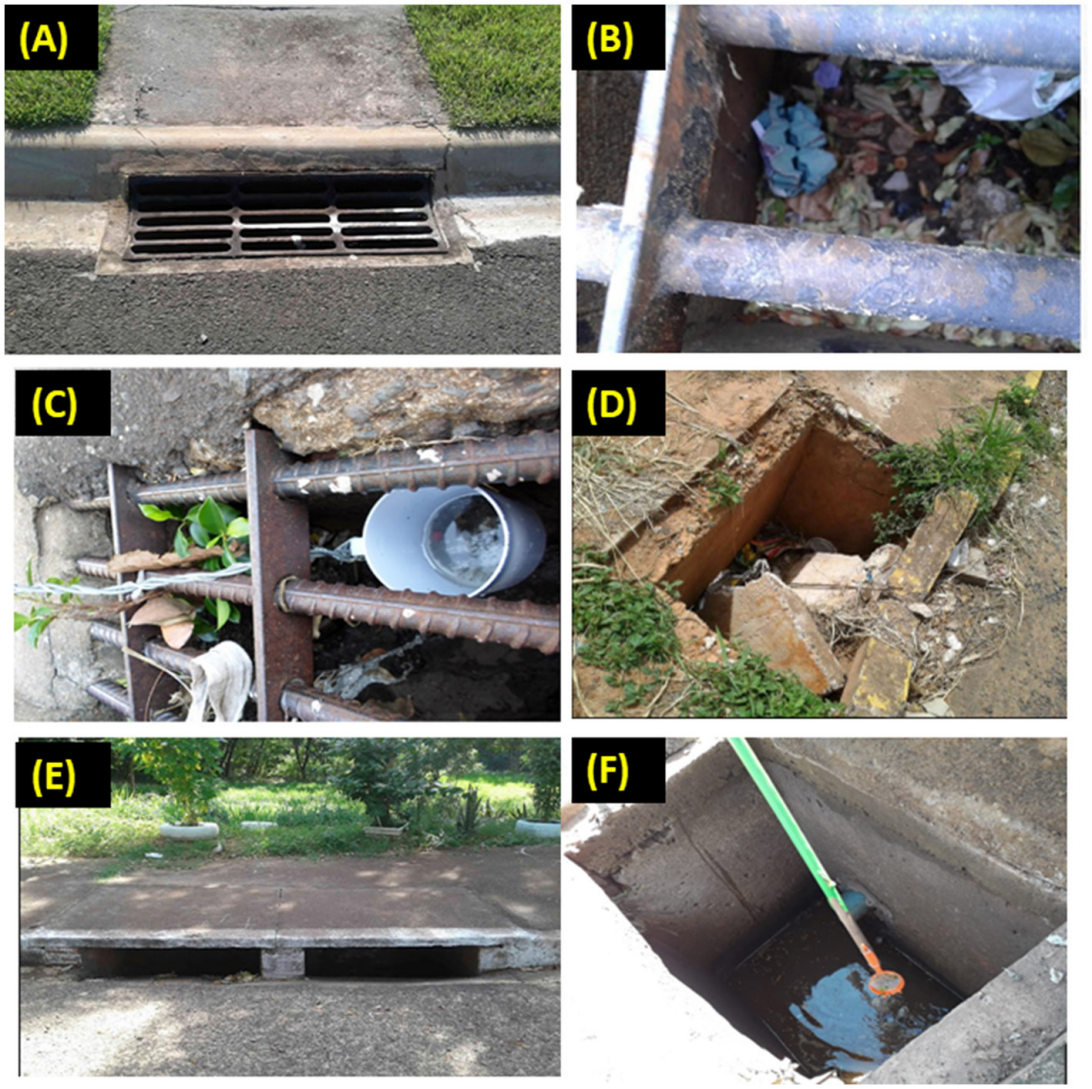
Examples of typical storm drains surveyed in the streets of Presidente Prudente, São Paulo, Brazil, between 2020 and 2021: **(A)** clean; **(B)** dirty; **(C)** clogged; **(D)** damaged; **(E)** not visible; **(F)** with water.

Trained technicians followed the map of the urban area of Presidente Prudente, identified the settings to be inspected, localized the storm drains, and filled in the spreadsheet with the following information: date of visit; visit time; latitude; longitude; area; sector; block number; storm drain number; internal and external temperature. *were classified into multiple categories* temperature was obtained using a digital thermometer with maximum and minimum measurements and an external sensor and alarm (Incoterm, Rio Grande do Sul, Brazil). To measure the internal temperature, the thermometer was inserted into the bottom of the storm drain and held for 2 min; for the external temperature, the thermometer was placed 2 cm from the surface of the storm drain and held for 2 min, then the measurements were recorded in a spreadsheet. Each storm drain was inspected at a single time point, i.e., only once, and classified according to the following criteria: (a) clean, with only running water and clearly visible; storm drains containing water were defined as those containing stagnant water; (b) dirty, containing discarded containers such as bottles, disposable cups, pieces of bicycle, dead animals, garbage bags, mattresses, plastic packaging, pieces of wood, construction debris, rotting food, and old shoes, among others; (c) clogged, containing construction materials, earth, and rubbish partially or completely obstructing the entrance; (d) damaged, the lid partially or completely broken; and (e) not visible (Figure 2). Some storm drains were classified into multiple categories: dirty, clogged, and damaged.

### 2.5 Adult *Ae. aegypti* collection using an aspirator inside the storm drains

Our field technician used a mechanical entomologic aspirator of Nasci, adapted by SUCEN to collect adult Ae. aegypti at specified storm drains within a 10-m radius. They started the collection from the right to the left, from up to down, respectively, at least 5 times. Holding the aspirator at a minimum 1-meter horizontal distance from the drain, the technician approached the drain and prepared for *Ae. aegypti* capture. Then they stomped on the drain to disturb resting *Ae. aegypti*, prompting them to take flight, while simultaneously using the aspirator to capture escaping *Ae. aegypti* or those resting nearby. Subsequently, the storm drain was opened and the interior space and walls were systematically vacuumed to capture any resting *Ae. aegypti*. If water was present in the drain, he aspirated the walls closest to the water because *Ae. aegypti* tend to rest in these areas. If there was no water, he aspirated the entire interior space of the storm drain to capture the *Ae. aegypti*. After collection, they were picked up with metal tweezers, transferred to Eppendorf vials containing 70% alcohol, and stored at −20°C until identification.

### 2.6 Collection of larvae and pupae inside the storm drains containing water

An entomologic cup was used to obtain samples of larvae and pupae inside the storm drains. Two dips were performed in each storm drain with water. The storm drains were visited at one single time point during each season of the year. When it rained, the technicians waited at least 2 days to collect the samples. After submerging the dipping tool (volume 0.5 L with a 1.5-m-long handle) vertically into the water, the tool was withdrawn and the water sample was poured into a plastic basin. Using pipettes, larvae or pupae were extracted and transferred into Eppendorf vials, which were stored at −20°C until identification. Each 2.0-mL vial contained 1.0 ml of the water collected, and up to 20/30 larvae were added to each vial. If necessary, an additional vial was used.

### 2.7 Identification of larvae, pupae, and adult *Ae. aegypti* collected from the storm drains

The Eppendorf tubes containing individual samples of larvae, pupae, and *Ae. aegypti* were identified by trained workers in the Entomology Laboratory of Presidente Prudente Surveillance, supervised by a biologist from the Entomology Laboratory of Faculty of Sciences, Letters, and Education from the Oeste Paulista University, Presidente Prudente, São Paulo (FACLEP-UNOESTE) and checked for species using a stereomicroscope (40–50 × magnification). Morphologic features were described in taxonomic keys (10, 11). All the classified samples were checked, and the identification was confirmed by specialized professionals from the Superintendency of Endemic Disease Control (SUCEN) of Presidente Prudente, a public health agency in the State of São Paulo responsible for controlling and identifying vectors of different diseases such as cutaneous leishmaniasis, visceral leishmaniasis, and dengue. They were classified into the order (Diptera), family (Culicidae), genus (*Aedes*), and species (*aegypti*), according to the classification of the Technical Standards Manual of the Brazilian Health Ministry (10) and the *Aedes aegypti* Entomologic Surveillance Guide (11).

### 2.8 Geospatial and multivariate analysis

Kernel density was used to show hotspots of dengue cases and the distribution of storm drains in Presidente Prudente. Kernel density calculates the density of point features, creating a raster output grid with the intensity of the phenomenon. The method uses a smoothly curved surface over each occurrence point, presenting higher values at the geolocation and diminishing in the opposite direction. This study used a pixel size output of 70 × 70 m. The density of each output raster cell (pixels) was calculated by adding the values of all surfaces in the kernel where they overlapped the center of the raster cell. An Excel spreadsheet containing the addresses (places of residence) of positive cases of dengue in 2021 in the urban area of Presidente Prudente was provided. This file was exported to GIS ArcGIS (ArcGIS 10.8 software) and the column of addresses was geocoded. The number of infected individuals and deaths was obtained from the Municipal Surveillance Department of Presidente Prudente, and the estimated number of people living in the city from 2015 to 2022 was obtained from IBGE.

The Band Collection Statistics tool in ArcGIS 10.8 provides statistics for the multivariate analysis of a set of raster layers and correlates two or more layers. When using the computational covariance and correlation matrices option, the covariance and correlation matrices are presented in tables, as are the basic statistical parameters such as the minimum, maximum, mean, and standard deviation values for each layer. The correlation matrices show the values of the correlation coefficients that depict the relationship between two sets of data. In the case of a set of raster layers, the correlation matrix presents the cellular values of one raster layer as they relate to the cellular values of another layer. The equation to calculate the correlation is as follows:


$$\text{Cor}{\text{r}}_{\text{ij}}=\frac{\text{Co}{\text{v}}_{\text{ij}}}{\delta_{\text{i}}\delta_{\text{j}}}$$

where *Corr*_*ij*_ is the correlation between layer *i* and layer *j, Cov*_*ij*_ is the covariance between layer *i* and layer *j*, δ_*i*_ is the standard deviation of layer *i*, and δ_*j*_ is the standard deviation of layer *j*.

The correlation ranges from +1 to −1. A positive correlation indicates a direct relationship between two layers, i.e., when the cellular values of one layer increase, the cellular values of another layer are also likely to increase. A negative correlation means that one variable changes inversely to the other. A correlation of zero means that two layers are independent of each other (5).

## 3 Results

### 3.1 Distribution of dengue cases in Presidente Prudente 2006–2023

**Figure 3:**
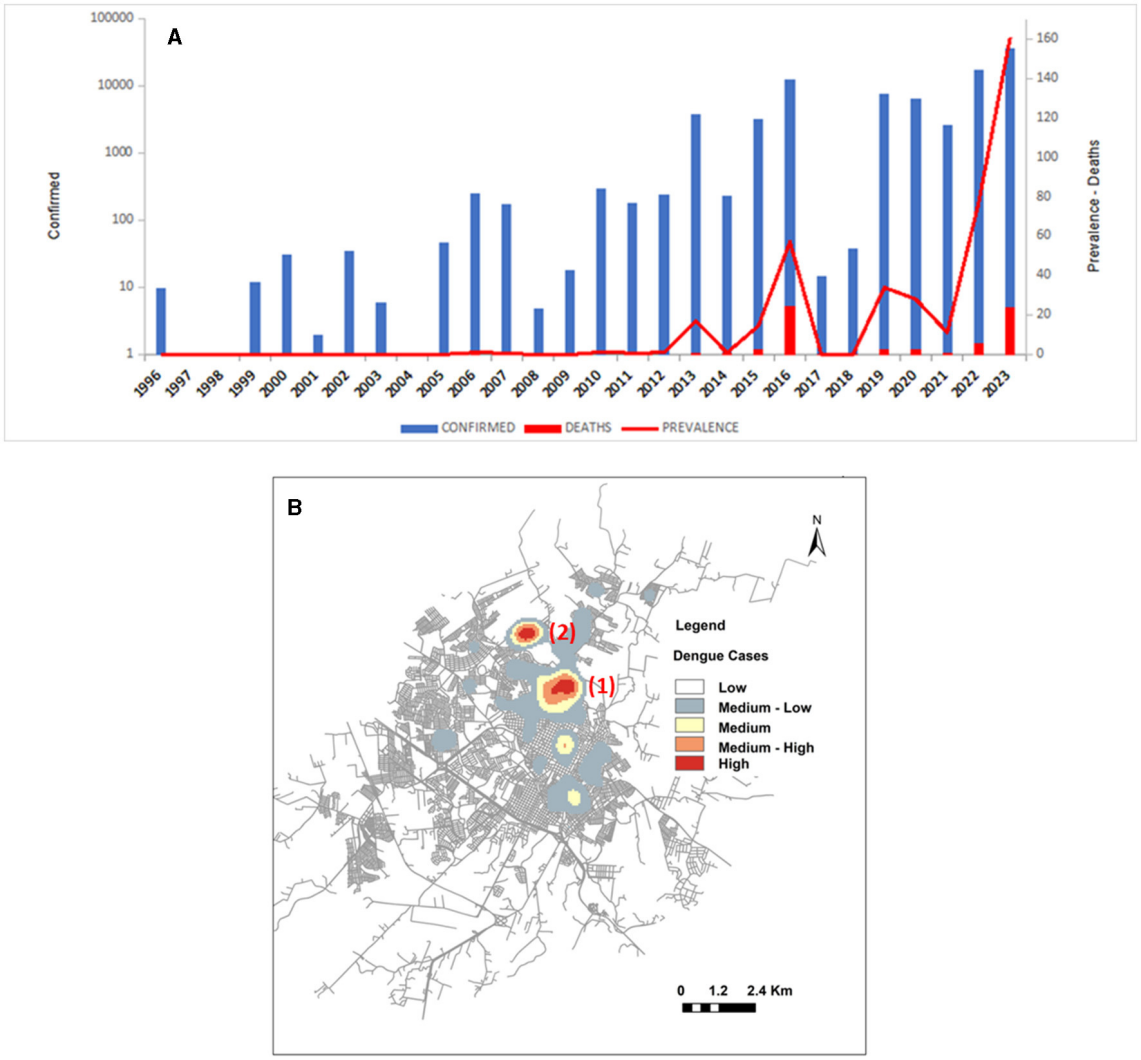
Epidemiology of dengue outbreaks in Presidente Prudente. **(A)** Total number of cases, deaths, and prevalence from1996 to 2023. **(B)** Estimated kernel density of notified cases of dengue in the urban area of Presidente Prudente (one point per patient) in 2021. Hotspots (1) and (2) represent areas where a higher density of notified cases was observed.

The outbreak of dengue in Brazil in 2023 changed from the northeast and central west region to São Paulo, in the southeast and to the Paraná state, in the south; Foz do Iguaçu and Maringa (Paraná) showed higher levels of cases nationwide, followed by Presidente Prudente in the western region of São Paulo state. The distribution of cases in Presidente Prudente between 1996 and 2023 has presented periods of low transmission interspersed with outbreaks. Cases as well as deaths peaked in 2016, slowed down, and returned to high levels in 2022 and increased again in 2023 (Figure 3A). Figure 3B shows the estimated kernel density of dengue cases in Presidente Prudente in 2021. Two thousand, one hundred and fifty cases were registered, and the clusters moved from area 04 in the eastern zone in 2020 to area 07 in the north in 2021, sectors 01 (163 cases) and sector 03 (255 cases) (Table 1).

### 3.2 Distribution and classification of storm drains in areas of Presidente Prudente

**Figure 4:**
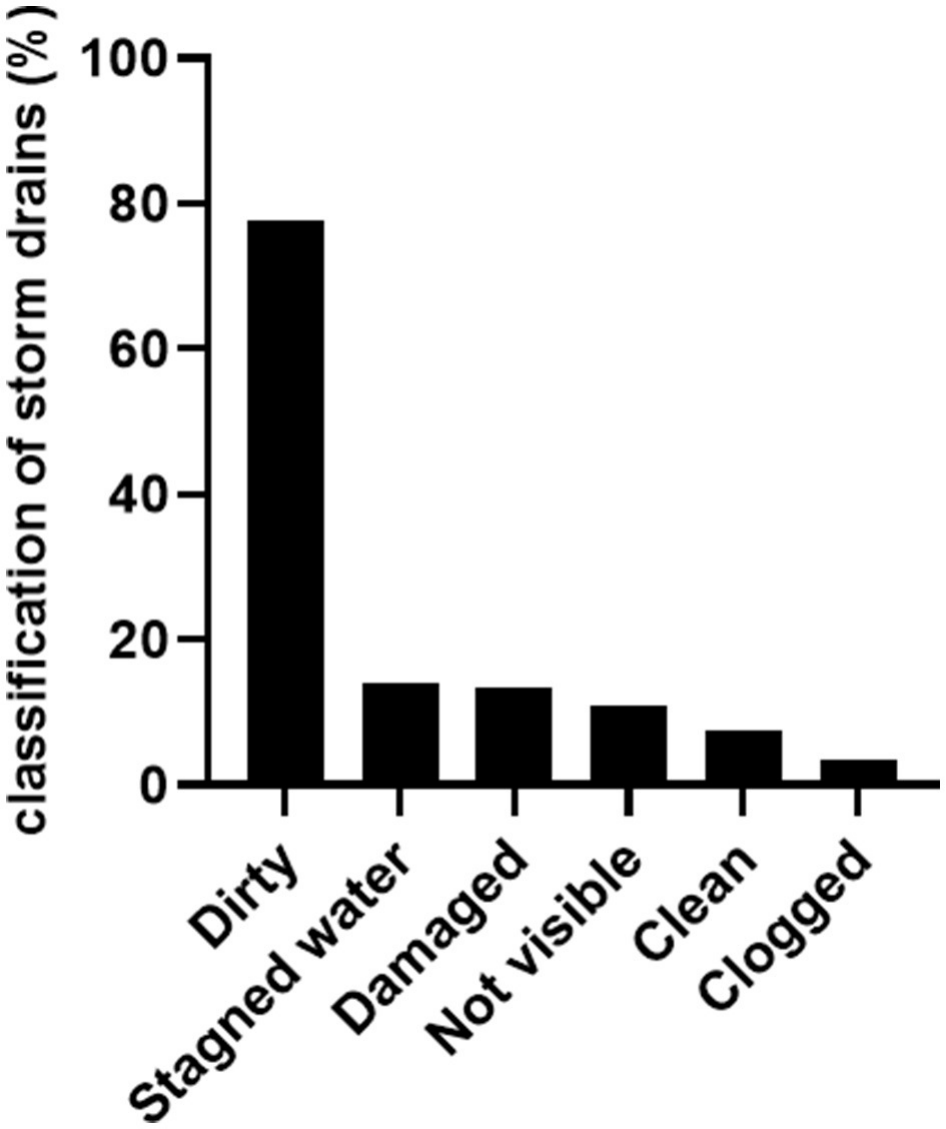
Bar plot representing the classification of storm drains found in different areas of Presidente Prudente.

Between 2020 and 2021, 5.492 storm drains were analyzed in six areas of Presidente Prudente (area 02 was not surveyed) [mean, 203.4 ± 25.14; interquartile range (IQR), 151.7–255.1]. A higher number of storm drains was found in area 04 (1,377 units), but higher means were found in areas 06 (286.8 ± 103.7; IQR, −43.16–616.7) and 07 (281.8 ± 37.99; IQR, 160.8–402.7). The number of storm drains in each area and sector is shown in Table 1. Of the 5,492 storm drains analyzed, 4,264 (77.6%) were classified as dirty, 777 (14.1%) had stagnant water, 735 (13.4%) were damaged, 602 (11.0%) were not visible, 404 (7.4%) were clean, and 195 (3.5%) were clogged (Figure 4). In some situations, a storm drain received multiple classifications.

### 3.3 Storm drains containing water in different areas of Presidente Prudente

**Figure 5:**
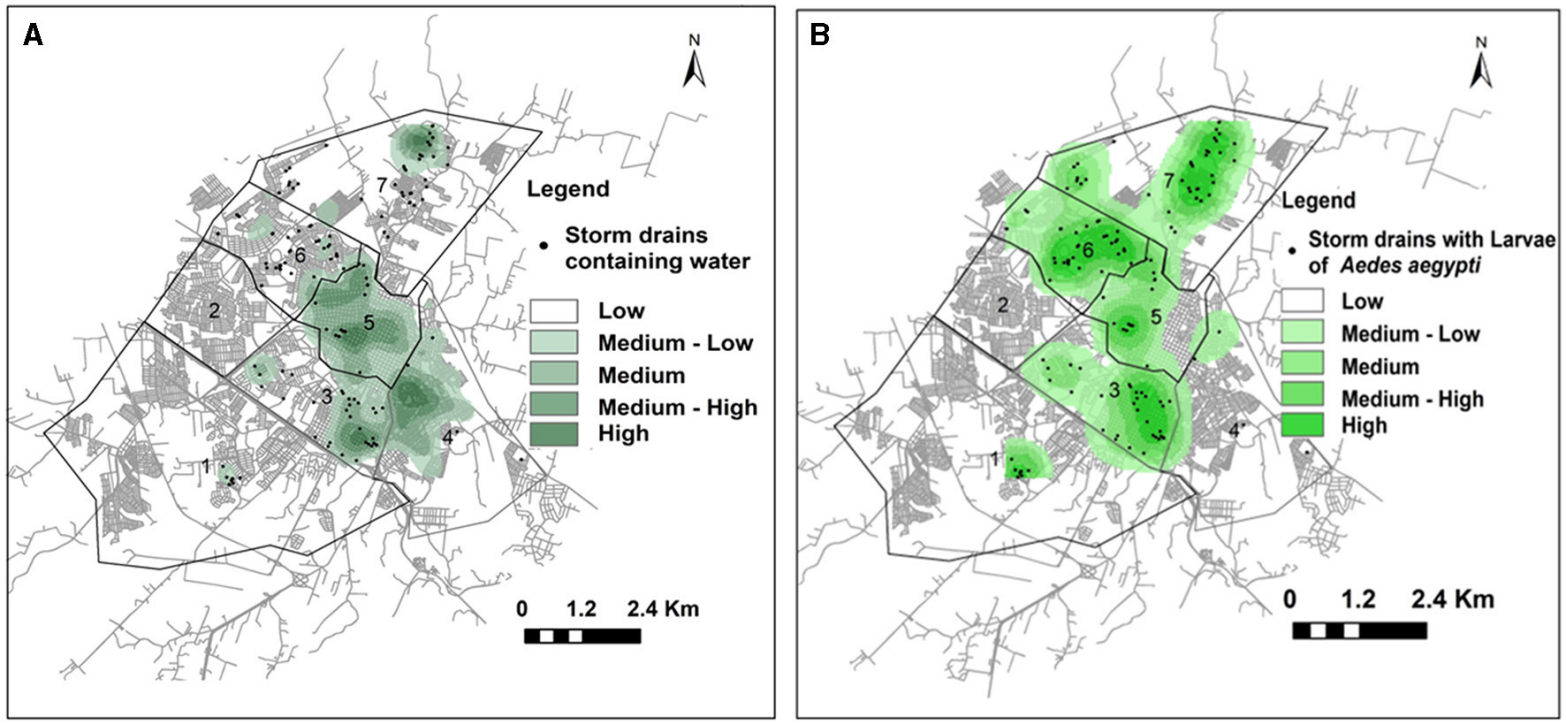
**(A)** Storm drains containing water (2020–2021), in the urban area of Presidente Prudente. The hotspots represent areas where a higher density of storm drains containing water was observed. The correlation between water in the storm drains and cases of dengue was *r* = 0.651. **(B)** Storm drains containing larvae/pupae of *Ae. aegypti* (2020–2021) in the urban area of Presidente Prudente. The hotspots represent areas where a higher density of storm drains containing larvae and dengue cases was observed. The correlation between the storm drains containing larvae and cases of dengue was *r* = 0.576.

Storm drains containing water are also a concern because they favor the development of pupae and larvae, as well as the adult *Ae. aegypti*. The water should have drained away completely. Of 5,492 storm drains, water was found in 777 (14.1%) distributed as follows: area 01, 10; area 03, 122; area 04, 212; area 05, 239; area 06, 83; and area 07, 111. Area 07 and sector 04 had a high number of storm drains containing water (61 units), and high and medium-high hotspots were also found. The kernel map shows that the high and medium-high hotspots of storm drains containing water were found in areas 04 and 05 (Table 1; Figure 5A).

### 3.4 The presence of larvae, pupae, and adult specimens of *Ae. aegypti* in storm drains

Concerning the presence of larvae and pupae of *Ae. aegypti* in different areas and sectors, higher means were found in areas 06 and 07 (8.75 ± 3.52; IQR, −2.45–19.95 and 9.75 ± 3.35; IQR, 0.91–30.41, respectively) (Table 1). Figure 5B shows the kernel map of storm drains with larvae and pupae. Of 777 storm drains distributed in the urban area of Presidente Prudente containing water, larvae/pupae of *Ae. aegypti* were found in 140 (18.0%) (Table 1). High and medium-high hotspots were found in sectors 2 of area 06 and sector 2 of area 07, where a high number of pupae and larvae was found (Table 1). Four adult specimens of *Ae. Aegypti*, three females and one male (one sample in each sector) were found in area 01, sector 04; area 03, sector 03; area 05, sectors 02 and 03.

### 3.5 Correlation between cases and the presence of water, larvae, and pupae in the storm drains

Using a geospatial approach, the kernel layer of cases of dengue diagnosed in 2021 was correlated with the kernel layer of storm drains containing water. The positive correlation between the water inside the storm drains and cases of dengue was *r* = 0.651, indicating a direct relationship between the two layers. In addition, the kernel layer of cases of dengue diagnosed in 2021 was correlated with the kernel layer of storm drains containing larvae and pupae of *Ae. aegypti* (*r* = 0.576), indicating a positive relationship between the two layers. To examine if other insights could be inferred from dengue cases, we looked at different combinations of storm drains, dirty, clogged, and damaged, and the correlations were 0.52, 0.51, and 0.41, respectively.

## 4 Discussion

The direct correlation between water in the storm drains and cases of dengue (*r* = 0.651), and water plus larvae and pupae of *Ae. aegypti* in the storm drains and cases of dengue (*r* = 0.576) suggest that the storm drain system is a risk factor for the maintenance of dengue endemicity and outbreaks in Presidente Prudente. As far as we know, using the kernel layer model, these results have not been shown previously and can be helpful mainly to public health policymakers to advance our understanding of dengue spreading in urban centers.

In 2023, Presidente Prudente, with a population of 225,688 inhabitants, had the third highest number of people infected with dengue in Brazil (36,197), which accounts for 16% of the entire population; furthermore, the city had the highest incidence of deaths in the country. This alarming number of people infected in 2023 needs to be analyzed considering risk factors that involve Latin American countries, and national and regional aspects. On a national scale, Brazil has distinct regions and biomes, resulting in social, environmental, and entomologic characteristics that support the spread of the *Ae. aegypti*. The effects of the El Nino phenomenon and climate change with intense droughts in the Amazon region and uncontrolled floods in the south, especially in light of the rising temperatures, favor the geographic spread of dengue vectors and expose new populations to dengue transmission (1, 4, 5). On a local scale, in the western region of São Paulo state, recent variations in precipitation patterns with droughts in the summer and rainfall in the winter have strongly impacted *Ae. aegypti* breeding sites, influencing the dynamics of dengue transmission (1, 2, 5). At national and regional levels an important risk factor is local communities' lack of engagement and mobilization in vector control activities including surveillance systems, integrated vector management, and health education (1, 4, 5). Despite the constant effort of public health authorities to control the vector, the population seems to ignore all the efforts, and in most Brazilian cities, from downtown to the outskirts, there are large areas of open-air garbage deposits. However, studies on the role of storm drains and the underground drainage system as risk factors for the maintenance of dengue endemicity are scarce, in Brazil and Latin American countries.

Clusters of dengue cases moved from area 04 in 2020 in the eastern region to area 07 in 2021 in the north, a vulnerable region with several risk factors for vector-borne diseases. The region has clusters with high and medium levels of cases; the region is located on the outskirts, 6.0 km from downtown, with a high number of vacant lots and non-urbanized areas. It is characterized by garbage scattered across the lots and a low-income population, who live mainly in the neighborhoods of two big housing complexes, Humberto Salvador and Augusto de Paula (5). These characteristics are different from area 04, where, for several years, clusters with higher levels of dengue cases were found (4, 5). Area 04 is also characterized by environmental and socioeconomic vulnerabilities, including high population density, a public landfill site near the urban area, forest fragments beside a river and its effluents, and a low-income population. It has been pointed out that, worldwide, mainly in tropical countries, a high incidence of dengue is linked to low income and socioeconomic status. Poverty could be related to factors that increase the risk of human exposure to *Ae. aegypti*, including lack of sanitation, low education, malnutrition, and housing deficiencies (12). In Presidente Prudente, it was demonstrated that a higher incidence of vectors and dengue cases occurred in the outskirts and low-income settings (5). The lessons learned from years of observing the constant movement of dengue outbreaks from one region to another are that preventive actions must be effective and long-lasting; otherwise, we will always be putting out fires. Area 04 had the highest number of storm drains in Presidente Prudente, and they certainly contributed to the permanence of the clusters of dengue in this setting in the last years.

In the urban area of Presidente Prudente, from 2020 to 2021, a high number of storm drains (5,492) were assessed, inspected, and classified. Higher levels were found in area 04, but higher means were found in areas 06 and 07. Unfortunately, many storm drains were damaged, compromising their water drainage function. The need for constant maintenance of the underground draining system by public authorities is well-known. In developing countries, particularly in Brazilian municipalities, storm drain management is not institutionalized, relies on municipal budget allocations, and does not have skilled staff (13). Stagnant water was found inside 14.1% of the storm drains, with clusters distributed mainly in areas 04, 05, and 07. All the water that runs from the streets into the storm drains must be drained immediately; if this does not happen, there is something wrong (13–16), correlating with the lack of infrastructure and maintenance of the drainage system. *Ae. aegypti* is a synanthropic species that uses stagnant water to complete its reproductive cycle (14–18). Irregular conditions in storm drains were also found in Merida, Mexico, and Salvador, Brazil (15, 18, 19). In Florida and neighboring states, storm drains are important reservoirs of mosquito species directly involved in the transmission of vector-borne diseases and they are likely to develop in large numbers in the nutrient- rich water trapped in storm drain basins (18). In Vero Beach and Key West, a significant number of *Ae. aegypti* and *A. albopictus* were found in storm drain basins (18). In the urban area of Guadalajara de Buga, southwestern Colombia, street storm drains were found to be a potential breeding site and to contain immature stages of *Aedes*; targeted control in these areas helped to decrease dengue transmission (20). Salvador, the capital of Bahia state, has some similarities with Presidente Prudente, including tropical climate, and poor neighborhoods in the periphery. It is considered endemic for dengue, and it was demonstrated that storm drains often accumulate water and serve as larvae development sites and adult resting areas for both *Ae. aegypti* and *A. albopictus* (17). The low number of adult specimens of *Ae. aegypti* was an intriguing finding. Following the protocol of the Technical Standards Manual Surveillance of Brazil's Ministry of Health (10), the aspirator was used first, and then the larvae and pupae were collected inside the storm drains. The noise from the aspirator may have scared away mainly *Ae. aegypti* adults as well as larvae and pupae.

The main strength of this study is the direct correlation between storm drains and dengue cases. Our data has global public health relevance, mainly in developing tropical countries sharing the socioeconomic and climate characteristics of Presidente Prudente.

Several shortcomings in our research must be mentioned. The storm drains were not standardized; some of them were very deep and some lids were fixed or had bars, which made access for evaluation and collection of larvae difficult. The high turnover of technicians made it difficult to standardize the visits and inspections of the storm drains. The entire area 02 was not surveyed as well as many sectors of areas 01 and 03. Another limitation was that the storm drains were visited in a single time point survey. These issues reflect the lack of infrastructure and maintenance of the underground drainage system in Presidente Prudente.

## 5 Conclusion

In Presidente Prudente, the incidence of dengue did not follow a cyclical behavior with periods of latency and outbreaks. In contrast, the distribution of dengue cases presented periods of low transmission interspersed with epidemics, and the city was considered hyperendemic in recent years. We found a direct correlation between the storm drains containing larvae and pupae of *Ae. aegypti* and cases of dengue, suggesting that storm drains are risk factors and have an impact on the maintenance of dengue endemicity in Presidente Prudente. Furthermore, the analysis showed a high number of damaged units, demonstrating a lack of storm drain management, compromising the urban drainage system. Policymakers may use these findings to improve existing dengue control strategies focusing on the control of storm drains and increasing local and global perspectives on reducing dengue outbreaks.

## Data Availability

The raw data supporting the conclusions of this article will be made available by the authors, without undue reservation.
